# Monitoring quality of obstetric care from hospital discharge databases: A Delphi survey to propose a new set of indicators based on maternal health outcomes

**DOI:** 10.1371/journal.pone.0211955

**Published:** 2019-02-12

**Authors:** Priscille Sauvegrain, Anne Alice Chantry, Coralie Chiesa-Dubruille, Hawa Keita, François Goffinet, Catherine Deneux-Tharaux

**Affiliations:** 1 Inserm UMR 1153, Obstetrical, Perinatal and Pediatric Epidemiology Research Team (Epopé) Center for Epidemiology and Statistics Sorbonne Paris Cité, DHU Risks in pregnancy, Paris Descartes University, Paris, France; 2 Department of Obstetrics and Gynecology, AP-HP Pitié-Salpêtrière, Paris, France; 3 School of Midwives, Baudelocque, AP-HP, University of Paris Descartes, DHU Risks in Pregnancy, Paris, France; 4 Department of Anesthesia and reanimation, AP-HP Louis Mourier, DHU Risks in Pregnancy, Colombes, France; 5 Paris Diderot university, Sorbonne Paris Cité, EA 7334 Recherche Clinique coordonnée ville-hôpital, Méthodologies et Société (REMES), Paris, France; 6 Department of Obstetrics and Gynecology, AP-HP Cochin-Port Royal, DHU Risks in Pregnancy, Paris, France; University of Sydney, AUSTRALIA

## Abstract

**Objectives:**

Most indicators proposed for assessing quality of care in obstetrics are process indicators and do not directly measure health effects, and cannot always be identified from routinely available databases. Our objective was to propose a set of indicators to assess the quality of hospital obstetric care from maternal morbidity outcomes identifiable in permanent hospital discharge databases.

**Methods:**

Various maternal morbidity outcomes potentially reflecting quality of obstetric care were first selected from a systematic literature review. Then a three-round Delphi consensus survey was conducted online from 11/2016 through 02/2017 among a French panel of 37 expert obstetricians, anesthetists-critical-care specialists, midwives, quality-of-care researchers, and user representatives. For a given maternal outcome, several definitions could be proposed and the indicator (i.e. corresponding rate) could be applied to all women or restricted to specific subgroup(s).

**Results:**

Of the 49 experts invited to participate, 37 agreed. The response rate was 92% in the second round and 97% in the third. Finally, a set of 13 indicators was selected to assess the quality of hospital obstetric care: rates of uterine rupture, postpartum hemorrhage, transfusion incident, severe perineal lacerations, episiotomy, cesarean, cesarean under general anesthesia, post-cesarean site infection, anesthesia-related complications, postpartum pulmonary embolism, maternal readmission and maternal mortality. Six were considered in specific subgroups, with, for example, the postpartum hemorrhage rate assessed among all women and also among women at low risk of PPH.

**Implications:**

This Delphi process enabled us to define consensually a set of indicators to assess the quality of hospital obstetrics care from routine hospital data, based on maternal morbidity outcomes. Considering 6 of them in specific subgroups of women is especially interesting. These indicators, identifiable through codes used in international classifications, will be useful to monitor quality of care over time and across settings.

## Introduction

For several years, safety at birth and the quality of care in the perinatal period have been a topic of concern to public health officials, care providers, and patient groups around the world. Accordingly, several sets of indicators assessing quality of care in obstetrics have been proposed but no consensus has emerged around any of them [1–11]. They have three principal limitations. First, many define the quality of care by indicators not directly associated with health (e.g. organization of care [5, 6] or process and practice [8, 9, 12]). Nonetheless, the final objective of evaluation of the quality of care is to improve health and reduce the frequency of adverse health outcomes. Another limitation of quality indicators based on health outcomes is that they often rely on vague or heterogeneous definitions that can lead to various interpretations. For example, some postpartum hemorrhage definitions are very precise (threshold of blood loss >1000 mL in the first 24 hours) [8], while others do not mention any threshold [4, 7]. Lastly, most indicators are defined in the general population of women giving birth and not assessed within specific subgroups where they could best reflect the quality of care.

Severe acute maternal morbidity (SAMM), associated with the most pathological forms of maternal health, complicates around 1% of deliveries. Its events are considered avoidable in a large fraction of cases, for reasons often linked to inadequate or faulty quality of care [13]. An evaluation tool based on the severe maternal morbidity outcomes that best reflect quality of care might therefore constitute a useful contribution to quality surveillance in obstetrics.

Permanent hospital discharge databases that synthesize information on each hospital stay exist in the great majority of high-resource countries, usually for initial billing purpose. They are coded in a standardized manner and contain diagnostic codes derived from the international ICD9 or ICD10 classifications used worldwide, and procedure codes derived from classifications that may be more country-specific but for which equivalences between countries can be established. They are therefore a potential common source for the continuous surveillance of quality of care in the great majority of high-income countries. In addition, two aspects of this data source are of particular interest for the identification of severe maternal outcomes. First, it allows exhaustive coverage, as mothers with serious complications are systematically hospitalized, in high-resource settings. Second, the validity of the reporting of some of these severe maternal outcomes in this database has already been assessed [14, 15].

The objective was to propose a set of indicators of the quality of hospital obstetric care chosen from severe maternal morbidity and mortality outcomes that reflect the quality of care, can be modified by improving practices, are reliably identifiable in routine hospital discharge data and do not depend exclusively on individual characteristics.

## Materials and methods

### Organization of the delphi process

We used a formal Delphi expert consensus method to reach a consensus in the selection of indicators to characterize the quality of hospital obstetric care. This method took place in several stages: i) first, we conducted a systematic review of the literature of the different indicators proposed internationally to assess the quality of obstetric care, ii) next, we set up a panel of French experts representing the specialists/professionals concerned: gynecologists-obstetricians, midwives, and anesthetists-critical-care specialists, together with representatives of service users, and iii) finally, we applied Delphi techniques to consult this panel. The process was supervised by a Scientific Committee composed of AAC, CCD, CDT, FG, HK and PS, who represented the professions consulted and are also researchers in epidemiology and social sciences or specialists in the quality of care.

This study was approved by the Advisory Committee on health research information (CCTIRS) on October 9, 2014, and by the CNIL (n° DR-2015-233, April 17, 2015, enabling us to use PMSI data). Because the Momassi project is not an interventional research and does not collect individual information, it did not require a formal ethics agreement, in accordance with the French law (Jardé legislation).

### Literature review

Based on a systematic review of the literature about the quality of obstetric care, we drafted an initial list of indicators. To review the literature, we searched PubMed with the key words "quality indicators" and "obstetrics" for the period beginning January 1, 2003, and ending August 31, 2016 (327 articles). Review of the bibliographies in these articles led to the addition of 17 more articles to the corpus.

A review of the abstracts of these 344 articles enabled us to classify 186 as not relevant, because they dealt with oncology, assisted reproductive technology, or gynecology. Another 42 articles concerned low-resource countries and were not transposable to settings in rich countries; 15 concerned solely fetal/neonatal outcomes, and 9 had either no abstract at all or no abstract in English or French.

**Fig 1:**
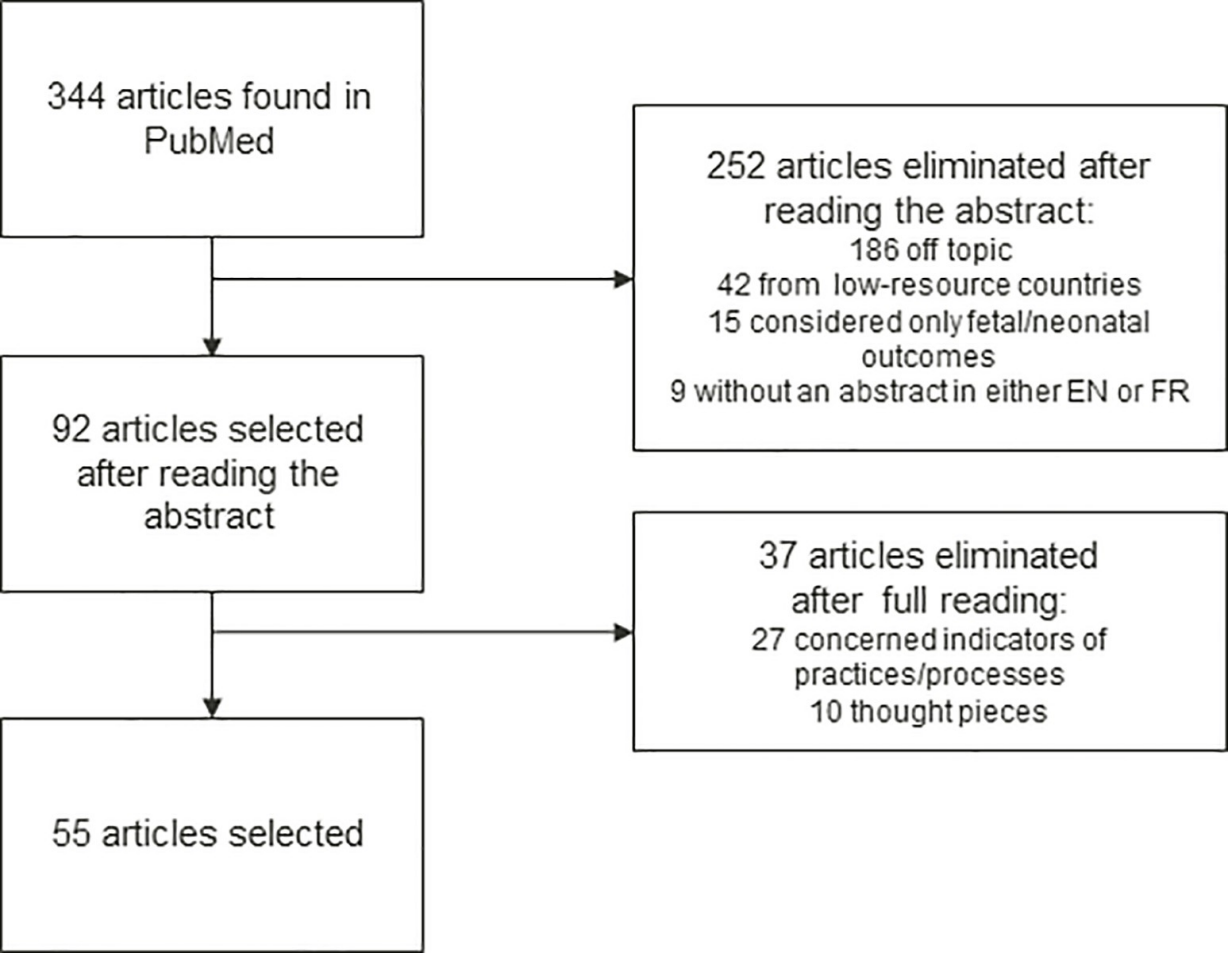
Momassi delphi–flow chart of articles selected.

We therefore selected 92 articles for a full reading and discussion by the Scientific Committee. After reading, 27 were eliminated from consideration because the quality indicators that they mentioned were indicators of practices, and 10 more articles because they were thought pieces about quality of care rather than analyses of its indicators. The remaining 55 articles mentioned indicators of quality of care based on maternal morbidity outcomes (listed in Appendix 1). This review of the literature finally produced 15 indicators that met the eligibility criteria we had defined: a maternal morbidity event proposed as a quality indicator and for which we had verified the availability in French routine hospital discharge data. Fig 1 synthesizes the process of the literature review and selection. The Prisma checklist is available in supporting information section (S1 File).Consultation of the websites and reports of various national and international learned societies did not identify any additional indicators not already mentioned in the literature we had reviewed.

### Constitution of a panel of experts

The experts recruited were either designated by three learned societies (the French National College of Gynecologists-Obstetricians, the National College of Midwives and the Club of Anesthetists-Critical-Care specialists in obstetrics) as experts in severe maternal morbidity or had worked on guidelines for the quality of care issued by French institutions (French National Authority for Health or the French National College of Gynecologists-Obstetricians). A balance between the various types of maternity units (of differing size and status) and the places (regions) of practice was sought. We also recruited user representatives through 3 associations of patients. Of the 49 individuals approached, 37 agreed to participate in this study. The panel included 16 obstetricians, 6 midwives, 11 anesthetists-critical-care specialists, 2 representatives of user associations, one specialist in the quality of care, and one person who preferred not to be identified.

### Organization of the consultation and analytic strategy

Initiated during the 1950s in the United States, the Delphi-type consensus approach makes it possible to organize a consultation of experts on a specific subject [16] by using iterative questionnaires (3 or 4 rounds); the panel must have feedback about all of the opinions stated to reach new positions, and the exchanges remain anonymous, which encourages free expression and avoids opinion leader effects. Delphi consensus processes have a proven track record for selecting indicators of quality of care in obstetrics [17]. This consultation was organized by a Delphi process in three rounds, completed online with Survey Monkey® polling software, from November 2016 through February 2017.

Each questionnaire was sent by individual emails. A reminder was sent to all participants 48 hours before the deadline, and another to those who had not responded at the deadline. The experts were to assess whether or not each of the 15 indicators was a good indicator of the quality of obstetric care, that is, was rarely expected except in cases of inadequate quality of care or variations of which reflected various levels of quality of care. The first questionnaire also proposed that the indicator be considered for some specific subgroups, because these morbid outcomes might reflect quality of care particularly when they occur among these subgroups, such as women in principle at low risk of the morbid complication considered. The experts were asked to assess the relevance of each indicator for this population (or these populations). It was clearly stated that the objective was not to combine the indicators selected into a global rate but rather to consider them one by one. Finally, the experts could suggest additional indicators if they wished.

Each of the proposed indicators was rated by the participants on a 4-point Likert-type scale: 1 = Disagree strongly, 2 = Disagree, 3 = Agree, and 4 = Agree strongly.

All of the analyses were conducted anonymously. For the first round, a threshold of 75% of consistent responses between the participants—of agreement or disagreement for each question (indicator, definition, or population)—was chosen to define consensus. Items scored as 1 and 2 were added together for disagreement and those coded scored 3 or 4 for agreement. For the second and third rounds, a threshold of 70% of consistent responses was chosen. To avoid a fourth round, the Scientific Committee decided to include indicators or populations very close to the threshold (>66%) in the third round. Those not reaching a consensus in the third round were not selected.

## Results

Among the panel of 37 participants in the first round, the participation rate was 92% in the second round (34 respondents) and 97% in the third (36 respondents).

Table 1 recapitulates the indicators submitted to the panel as well as the precisions of definitions or groups of women concerned, and those selected at the different rounds.

**Table 1:** Momassi delphi process: Indicators of quality of care considered and selected or excluded at different rounds of the consultation.

Indicators	Consensus to include or exclude the indicator	Agreement % (Round)	Among the indicators included details for the definition (D_) or population (P_) *and I_ subindicators of complications of anesthesia*	Final decision	Agreement % (Round)
**Uterine rupture rate**	Included	77% (1)	P_ Among women with a previous cesarean	Included	77% (1)
			P_ Among all women	Excluded	76% (1)
**Postpartum hemorrhage rate**	Included	84% (1)	D_ Any one or more of conservative surgery, embolization, hysterectomy or transfusion of 4 or more units of packed red blood cells	Included	88% (2)
			P_ Among all women	Included	79% (3)
			P_ Among women who had a planned cesarean before labor	Excluded	93% (1)
			P_ Among women at low risk of PPH*	Included	66% (3)
**Maternal transfusion complication rate**	Included	90% (1)			
**Severe perineal laceration rate**^**1**^	Included	72% (2)	D_ Third- and fourth-degree lacerations	Included	80% (2)
			P_ Among all women	Included	74% (2)
			P_ Among women with a spontaneous vaginal delivery	Included	69% (3)
			P_ Among women with a non-macrosomic fetus	Not selected	45% (3)
**Episiotomy rate**^**1**^	Included	83% (1)	P_ Among all women	Included	73% (1)
			P_ Among women with a spontaneous vaginal delivery	Included	66% (3)
			P_ Among women with a non-macrosomic fetus	Not selected	44% (3)
			P_ Among multiparous women	Not selected	50% (3)
**Cesarean rate**	Included	94% (1)	P_ Among all women	Included	69% (3)
			P_ Among women at low risk of cesarean**	Included	77% (2)
			P_ Before labor, among women at low risk of cesarean**	Included	69% (3)
**Rate of cesareans under general anesthesia during labor**	Included	75% (3)			
**Post-delivery laparotomy rate**	Not selected	44% (3)			
**Post-cesarean infection rate**	Included	95% (1)	D_ Only surgical site infections, including endometritis	Included	94% (3)
			D_ All infections	Not selected	31% (3)
			D_ Only wound/scar infections	Excluded	77% (2)
**Maternal pyelonephritis rate**	Excluded	89% (1)			
**Postpartum pulmonary embolism rate**	Included	76% (2)	P_ Among all women	Included	91% (3)
			P_ Among women with a planned cesarean	Not selected	56% (3)
**Eclampsia rate**	Not selected	59% (3)			
**Maternal readmission rate after hospitalization for delivery**	Included	89% (1)	D_ In the 42 days postpartum	Included	71% (2)
			P_ Among all women	Included	78% (2)
			P_ Among all women without complications of breast feeding	Not selected	41% (3)
**Maternal ICU admission rate**	Not selected	44% (3)			
**Maternal mortality rate**	Included	81% (1)	P_ All women	Included	84% (2)
			P_ Among women at low risk of maternal mortality***	Included	66% (3)
**Complications of anesthesia:** ***6 subindicators first specified in the second round***	Included	84% (1)	*I_ Rate of headaches induced by spinal or epidural anesthesia and for which a blood patch was performed*	Included	85% (2)
			*I_ Rate of anesthesia-related pulmonary aspiration*	Included	91% (2)
			*I_ Other anesthesia-related pulmonary complications*	Included	71% (2)
			*I_ Anesthesia-related cardiac complications*	Not selected	56% (3)
			*I_ Anesthesia-related complications involving the central nervous system*	Not selected	65% (3)
			*I_ Toxic reaction to local anesthesia *	Not selected	60% (3)
**Rate of other complications resulting from care**	Include	69% (3)			

^1^ The panel decided that these indicators should be monitored concomitantly rather than separately

* Population at low risk of PPH: singleton at term, with no abnormal placental insertion, no history of cesarean, and no preeclampsia

**Population at low risk of cesarean: singleton in cephalic presentation at term, no abnormal placental insertion, no history of cesarean, and no preeclampsia

***: Population at low risk of maternal mortality: singleton at term, no abnormal placental insertion, no history of cesarean, no preeclampsia, and no chronic maternal disease

The rates of uterine rupture (77% agreement), postpartum hemorrhage (84%), transfusion incidents (90%), third- and fourth-degree perineal tears or lacerations (80%), episiotomies (83%), cesareans (94%), post-cesarean infections (95%), complications from anesthesia (84%), maternal readmission post-delivery (87%), and maternal mortality (81%) were the indicators most frequently selected in the first round.

The panel excluded four indicators during the Delphi process. These were the rates of maternal pyelonephritis (89% agreement to exclude it in round 1), laparotomy after delivery (consensus not reached, exclusion at the end of round 3), eclampsia (consensus not reached, excluded at the end of round 3), and maternal ICU admissions. The latter had the particularity of receiving its highest vote count during the first round rather than any of the following rounds (not selected in the third round, with only 44% agreement). This change resulted from feedback from experts, who insisted that this indicator depends strongly on the local organization of care

Some indicators required adjustments. Accordingly, a proposal that the indicators of the rates of severe perineal lacerations and of episiotomies should be monitored concomitantly rather than separately was submitted to the panel and approved. To specify that it is most relevant in particular populations, it was finally decided to consider the cesarean rate in 3 different populations: among all women, among those at low risk of cesarean (singleton in cephalic presentation at term, with no abnormal placental insertion, no history of cesarean, and no preeclampsia) and among women who were at low risk of a cesarean but nonetheless had a cesarean before labor. These populations were specified only during the second and third rounds of the consultation.

At the conclusion of the first round, the panel considered that the indicator concerning the complications of anesthesia was too unspecific. The complications were then separated into subindicators, three of which were selected during the consensus process: headaches induced by spinal or epidural anesthesia and for which a blood patch was performed, anesthesia-related pulmonary aspiration, and "other pulmonary complications" of anesthesia. Moreover, the panel proposed 13 additional indicators. The Scientific Committee endorsed two of them because they concerned maternal health, were proposed by at least 2 experts, and could be identified in the hospital data. These were the rate of cesareans under general anesthesia and an item entitled "other complications associated with care," to be entered by medical staff at discharge, when applicable. Both of these received consensus approval in the second round. The other indicators proposed were not selected, because they concerned either neonatal health status or items related to practices rather than health status.

The restriction of indicators to one or several groups of women in which they might be considered more pertinent than in the general population was suggested for eight of the indicators proposed to the panel, and finally kept for six. A consensus was reached for 3 subgroups of women at the first round, rate of uterine rupture among women with a previous cesarean (77% agreement, note this is the only subgroup of women at higher risk than the general population), rate of episiotomy among all women (73% agreement), and rate of postpartum hemorrhage among women who had a planned cesarean before labor (93% agreement to exclude it in round 1).

The third round of consultation finally resulted in a set of 13 indicators of the quality of hospital care, reported in Table 2.

**Table 2:** The set of indicators of the quality of hospital obstetric care determined in the delphi process.

Groups of women concerned *Indicators selected*	All women	Particular subgroup of women		Recommanded surveillance period (postpartum = within 42 days)
**1. Uterine rupture rate**		Women with a previous cesarean		Antenatal Birth
**2. Rate of postpartum hemorrhage treated by one of more of embolization, surgery, or transfusion of more than four units of packed red blood cells** *		Women at low risk of PPH**		Postpartum
**3. Maternal transfusion incident rate**				Antenatal Birth Postpartum
**4. Rate of severe perineal lacerations (third- and fourth degree)**		Women with a spontaneous vaginal delivery		Birth
**5. Episiotomy rate**		Women with a spontaneous vaginal delivery		Birth
**6. Cesarean rate**		Women at low risk of cesarean***	Women at low risk of cesarean*** and with a cesarean before labor	Birth
**7. Rate of cesarean deliveries under general anesthesia during labor**				Birth
**8. Rate of post-cesarean surgical site infections, including endometritis**				Postpartum
**9A. Rate of headaches induced by spinal or epidural anesthesia and for which a blood patch was performed**				Postpartum
**9B. Rate of anesthesia-related pulmonary aspiration**				Birth Postpartum
**9C. Rate of other anesthesia-related pulmonary complications **				Birth Postpartum
**10. Rate of other complications resulting from care **				Antenatal Birth Postpartum
**11. Postpartum pulmonary embolism rate**				Postpartum
**12. Maternal readmission rate after postpartum discharge, within 42 days**				Postpartum
**13. Maternal mortality rate**		Women at low risk of mortality****		Antenatal Birth Postpartum

Gray boxes = selected groups of women

* Item reflecting "major" transfusion as coded in the French hospital discharge database

** Population at low risk of PPH: singleton at term, no abnormal placental insertion anomaly, no history of cesarean, and no preeclampsia

***Population at low risk of cesarean: singleton in cephalic presentation at term, no abnormal placental insertion, no history of cesarean, and no preeclampsia

****: Population at low risk of maternal mortality: singleton at term, no abnormal placental insertion, no history of cesarean, no preeclampsia, and no chronic maternal disease

## Discussion

### Principal results

Through a Delphi-type process that included a national panel of French experts, it was possible to define a set of 13 indicators to characterize the quality of hospital obstetric care chosen from maternal morbidity outcomes that can be identifiable in routine hospital discharge databases. One original aspect of the process was to consider assessing some indicators in particular subgroups of women in whom these outcomes are rarely expected; this choice makes it possible to identify perspectives to improve clinical practices.

### Strengths and limitations

The panel had thorough knowledge of the topic of quality of care and of maternal morbidity and represented 4 professions and different modes of practice. The excellent participation rate is evidence simultaneously of good panel selection, interest in the subject, and an effective email reminder strategy. Participation by users' representatives was one of the Scientific Committee's priorities and was achieved, although their participation in all three rounds of the consultation is rare in Delphi processes.

Although several of the sets of indicators of quality of care found in the literature include some components of maternal morbidity, they are generally mixed within composite indicators that include indicators of practices, organization of care, and/or neonatal outcomes [5–9]. To our knowledge and based on our systematic review of the literature on this topic, this set of indicators is the first that considers only indicators based on maternal morbidity outcomes. The combination of indicators assessing the quality of obstetric and anesthetic care in the same tool is also a strength and highlights the multidisciplinary approach to the management of obstetrical patients.

The choice of indicators identifiable in routine hospital discharge databases will allow regional and national comparisons and benchmarking of performance as well as ongoing surveillance of changes over time. The "diagnosis" indicators chosen in this process (e.g. uterine rupture, postpartum hemorrhage, perineal laceration, infection) are defined with precision by the panel and can be described by their codes in ICD9 or ICD10, the classifications used for coding medical information worldwide. They should accordingly enable international comparisons of these quality of care indicators. Finally, our proposal to consider some quality indicators in one or more specific subgroup of women is an originality of this project and suggests the utility of developing research in this direction.

This method of consultation nonetheless has limitations. Even though the panel was selected mainly by nominations from learned societies who identified experts on the subject matter, some respondents nonetheless found it difficult lend themselves entirely to this exercise without a feeling of defensiveness about their practices. Moreover, the consensus process led investigators/respondents to introduce components on the frontier between practices and maternal morbidity. This explains the less than severe character of some of the events included in the final set of indicators. This is also related to the well-known difficulty of defining a threshold or borderline for SAMM [18]. Furthermore, the choice of indicators by the panel may have been different in another national context; however, this appears unlikely since the main causes of maternal mortality are similar in France and in other high-income countries [19]. Finally, this set of indicators is not intended to cover all types of obstetric care but rather to focus mainly on delivery and its immediate consequences, although two indicators do explore the 42-day postpartum period (maternal readmission rate after hospitalization for delivery and maternal mortality rate). Indicators for the quality of prenatal care are also necessary and can be built applying the same procedure but must also include aspects of outpatient care and therefore other data sources.

While we have proposed a tool to assess the quality of hospital obstetric care with indicators available in hospital discharge databases, the translation of this set into an algorithm of codes will be followed by a validation stage. The validity of the reporting in hospital databases of some of the selected maternal outcomes has already been assessed, in France and internationally [14, 15, 20, 21]. We will complete this assessment through a comparison with data from specific French epidemiological population-based studies. It is also important to note that we have not assigned specific targets or thresholds to each event. This is probably the most complicated aspect of performance monitoring and could be considered in subsequent research.

### The participating clinicians and the quality of care

As Bailit et al. recently underlined in a recent SMFM report, the presence of clinicians within the research teams and expert groups working on indicator development, for example, both organizing and participating in Delphi procedures, is essential, to avoid misinterpretations that literature review alone can induce [22]. Nonetheless, quality procedures are more often perceived as coercive or constraining than as steps leading to favorable changes in outcomes, and a "culture of quality" or a quality mindset does not yet appear to be fully integrated into the culture of French hospitals. In the comment areas available, some respondents expressed reactions expressing an analysis based more on clinical reasoning than on quality of care. Accordingly, about the cesarean indicator, one obstetrician remarked: "I don't really understand the question, if there are fetal heart rate abnormalities [in a woman at] low risk of cesarean delivery, the cesarean is not an indicator of inadequate quality of care" (excerpt from questionnaire O16). Another obstetrician said about the episiotomy indicator: "I do not understand how an episiotomy constitutes defective care" (excerpt from questionnaire O20).

These comments point to the physicians' feelings of loss of autonomy, in response to the demands they face to standardize their practices, as Eliot Freidson's work shows [23].

### Point of discussion about the delphi method

Over the course of this Delphi consultation, some of the respondents' comments expressed their progressive ownership of the concept that some indicators can be usefully considered in some specific contexts or populations. This led us to wonder about the need for a conference (virtual or in person) to explain and train participants in the process before it starts. The Delphi method normally foresees, at least in one of its branches, the Delphi-Rand method, a final meeting to reach consensus. A preliminary training meeting may have two disadvantages: eliminating the panel members' anonymity and thus promoting opinion leader effects. It might also, however, have the advantage of clarifying the objectives.

## Conclusion

The consultation of clinicians who were also experts in quality of care, representing the professions involved, as well as user representatives, enabled us to create a set of indicators to assess the quality of obstetric care from hospital discharge databases. The set is composed of 13 indicators, including 6 which will be considered in subgroups of women. The translation of this tool into an algorithm of codes will be followed by a validation stage.

This set is composed of outcomes identifiable in routine databases through codes available in international classifications; it may then be applied across or within countries to reveal themes for improving obstetric care.

## Appendix 1: list of articles selected through the review of the international literature

Asch DA, Nicholson S, Srinivas S, Herrin J, Epstein AJ. Evaluating obstetrical residency programs using patient outcomes. Jama. 2009 Sep 23;302(12):1277–83.

Bahrami S, Holstein J, Chatellier G, Le Roux YE, Dormont B. [Using administrative data to assess the impact of length of stay on readmissions: study of two procedures in surgery and obstetrics]. Revue d'epidemiologie et de sante publique. 2008 Apr;56(2):79–85.

Bailit JL, Garrett JM. Stability of risk-adjusted primary cesarean delivery rates over time. American journal of obstetrics and gynecology. 2004 Feb;190(2):395–400.

Bailit JL, Gregory KD, Srinivas S, Westover T, Grobman WA, Saade GR. Society for Maternal-Fetal Medicine (SMFM) Special Report: Current approaches to measuring quality of care in obstetrics. American journal of obstetrics and gynecology. 2016 Sep;215(3):B8-B16.

Bailit JL, Grobman WA, Rice MM, Spong CY, Wapner RJ, Varner MW, et al. Risk-adjusted models for adverse obstetric outcomes and variation in risk-adjusted outcomes across hospitals. American journal of obstetrics and gynecology. 2013 Nov;209(5):446 e1- e30.

Bamfo JE. Managing the risks of sepsis in pregnancy. Best practice & research Clinical obstetrics & gynaecology. 2013 Aug;27(4):583–95.

Blondel B, Alexander S, Bjarnadottir RI, Gissler M, Langhoff-Roos J, Novak-Antolic Z, et al. Variations in rates of severe perineal tears and episiotomies in 20 European countries: a study based on routine national data in Euro-Peristat Project. Acta obstetricia et gynecologica Scandinavica. 2016 Jul;95(7):746–54.

Bloom SL, Spong CY, Weiner SJ, Landon MB, Rouse DJ, Varner MW, et al. Complications of anesthesia for cesarean delivery. Obstetrics and gynecology. 2005 Aug;106(2):281–7.

Bonfill X, Roque M, Aller MB, Osorio D, Foradada C, Vives A, et al. Development of quality of care indicators from systematic reviews: the case of hospital delivery. Implementation science: IS. 2013;8:42.

Boulkedid R, Sibony O, Bossu-Salvador C, Oury JF, Alberti C. Monitoring healthcare quality in an obstetrics and gynaecology department using a CUSUM chart. BJOG: an international journal of obstetrics and gynaecology. 2010 Sep;117(10):1225–35.

Boulkedid R, Sibony O, Goffinet F, Fauconnier A, Branger B, Alberti C. Quality indicators for continuous monitoring to improve maternal and infant health in maternity departments: a modified Delphi survey of an international multidisciplinary panel. PloS one. 2013;8(4):e60663.

Callaghan WM, Grobman WA, Kilpatrick SJ, Main EK, D'Alton M. Facility-based identification of women with severe maternal morbidity: it is time to start. Obstetrics and gynecology. 2014 May;123(5):978–81.

Commission. TJ. America’s hospitals: improving quality and safety. Available at: http://goo.gl/rrZBl6.: 2014 Retrieved August 29, 2016 Report

Coonrod DV, Drachman D, Hobson P, Manriquez M. Nulliparous term singleton vertex cesarean delivery rates: institutional and individual level predictors. American journal of obstetrics and gynecology. 2008 Jun;198(6):694 e1-11; discussion e11.

Dietz HP, Pardey J, Murray H. Pelvic floor and anal sphincter trauma should be key performance indicators of maternity services. International urogynecology journal. 2015 Jan;26(1):29–32.

Fantini MP, Stivanello E, Frammartino B, Barone AP, Fusco D, Dallolio L, et al. Risk adjustment for inter-hospital comparison of primary cesarean section rates: need, validity and parsimony. BMC health services research. 2006;6:100.

Florea A, Caughey SS, Westland J, Berckmans M, Kennelly C, Beach C, et al. The Ottawa hospital quality incident notification system for capturing adverse events in obstetrics. Journal of obstetrics and gynaecology Canada: JOGC = Journal d'obstetrique et gynecologie du Canada: JOGC. 2010 Jul;32(7):657–62.

Foglia LM, Nielsen PE, Hemann EA, Walker S, Pates JA, Napolitano PG, et al. Accuracy of the Adverse Outcome Index: An Obstetrical Quality Measure. Joint Commission journal on quality and patient safety / Joint Commission Resources. 2015 Aug;41(8):370–7.

Friedman AM, Phipps MG, Raker CA, Anderson BL. Pyelonephritis during pregnancy as a marker for quality of prenatal care. The journal of maternal-fetal & neonatal medicine: the official journal of the European Association of Perinatal Medicine, the Federation of Asia and Oceania Perinatal Societies, the International Society of Perinatal Obstet. 2012 Jun;25(6):739–42.

Gibson K, Bailit JL. Cesarean delivery as a marker for obstetric quality. Clinical obstetrics and gynecology. 2015 Jun;58(2):211–6.

Gilbert WM, Bliss MC, Johnson A, Farrell W, Gregg L, Swanson C. Improving recording accuracy, transparency, and performance for obstetric quality measures in a community hospital-based obstetrics department. Joint Commission journal on quality and patient safety / Joint Commission Resources. 2013 Jun;39(6):258–66.

Gregory KD, Korst LM, Lu MC, Fridman M. AHRQ patient safety indicators: time to include hemorrhage and infection during childbirth. Joint Commission journal on quality and patient safety / Joint Commission Resources. 2013 Mar;39(3):114–22.

Grobman WA, Feinglass J, Murthy S. Are the Agency for Healthcare Research and Quality obstetric trauma indicators valid measures of hospital safety? American journal of obstetrics and gynecology. 2006 Sep;195(3):868–74.

Grunebaum A. Error reduction and quality assurance in obstetrics. Clinics in perinatology. 2007 Sep;34(3):489–502.

Guglielminotti J, Li G. Monitoring Obstetric Anesthesia Safety across Hospitals through Multilevel Modeling. Anesthesiology. 2015 Jun;122(6):1268–79.

Haller G, Camparini-Righini N, Kern C, Pfister RE, Morales M, Berner M, et al. [Patient safety indicators for obstetrics: a Delphi based study]. Journal de gynecologie, obstetrique et biologie de la reproduction. 2010 Sep;39(5):371–8.

Howell EA, Zeitlin J, Hebert PL, Balbierz A, Egorova N. Association between hospital-level obstetric quality indicators and maternal and neonatal morbidity. Jama. 2014 Oct 15;312(15):1531–41.

Janakiraman V, Ecker J. Quality in obstetric care: measuring what matters. Obstetrics and gynecology. 2010 Sep;116(3):728–32.

Johnson CE, Handberg E, Dobalian A, Gurol N, Pearson V. Improving perinatal and neonatal patient safety: The AHRQ patient safety indicators. The Journal of perinatal & neonatal nursing. 2005 Jan-Mar;19(1):15–23.

Kesmodel US, Jolving LR. Measuring and improving quality in obstetrics—the implementation of national indicators in Denmark. Acta obstetricia et gynecologica Scandinavica. 2011 Apr;90(4):295–304.

Korst LM, Fridman M, Friedlich PS, Lu MC, Reyes C, Hobel CJ, et al. Hospital rates of maternal and neonatal infection in a low-risk population. Maternal and child health journal. 2005 Sep;9(3):307–16.

Korst LM, Reyes C, Fridman M, Lu MC, Hobel CJ, Gregory KD. Gestational pyelonephritis as an indicator of the quality of ambulatory maternal health care services. Obstetrics and gynecology. 2006 Mar;107(3):632–40.

Kristensen S, Mainz J, Bartels P. Selection of indicators for continuous monitoring of patient safety: recommendations of the project 'safety improvement for patients in Europe'. International journal for quality in health care: journal of the International Society for Quality in Health Care / ISQua. 2009 Jun;21(3):169–75.

Kyser KL, Lu X, Santillan DA, Santillan MK, Hunter SK, Cahill AG, et al. The association between hospital obstetrical volume and maternal postpartum complications. American journal of obstetrics and gynecology. 2012 Jul;207(1):42 e1-17.

Main EK, Moore D, Farrell B, Schimmel LD, Altman RJ, Abrahams C, et al. Is there a useful cesarean birth measure? Assessment of the nulliparous term singleton vertex cesarean birth rate as a tool for obstetric quality improvement. American journal of obstetrics and gynecology. 2006 Jun;194(6):1644–51; discussion 51–2.

Mann S, Pratt S, Gluck P, Nielsen P, Risser D, Greenberg P, et al. Assessing quality obstetrical care: development of standardized measures. Joint Commission journal on quality and patient safety / Joint Commission Resources. 2006 Sep;32(9):497–505.

Melman S, Schoorel EC, de Boer K, Burggraaf H, Derks JB, van Dijk D, et al. Development and Measurement of Guidelines-Based Quality Indicators of Caesarean Section Care in the Netherlands: A RAND-Modified Delphi Procedure and Retrospective Medical Chart Review. PloS one. 2016;11(1):e0145771.

Melman S, Schoorel EN, Dirksen C, Kwee A, Smits L, de Boer F, et al. SIMPLE: implementation of recommendations from international evidence-based guidelines on caesarean sections in the Netherlands. Protocol for a controlled before and after study. Implementation science: IS. 2013;8:3.

Murphy PA, Fullerton JT. Development of the Optimality Index as a new approach to evaluating outcomes of maternity care. Journal of obstetric, gynecologic, and neonatal nursing: JOGNN / NAACOG. 2006 Nov-Dec;35(6):770–8.

Neuman MD, Wirtalla C, Werner RM. Association between skilled nursing facility quality indicators and hospital readmissions. Jama. 2014 Oct 15;312(15):1542–51.

Norum J, Heyd A, Hjelseth B, Svee T, Murer FA, Erlandsen R, et al. Quality of obstetric care in the sparsely populated sub-arctic area of Norway 2009–2011. BMC pregnancy and childbirth. 2013;13:175.

Pakkanen M, Lindblom B, Olausson PO, Rosen M. [Great regional differences in quality of obstetrical care]. Lakartidningen. 2004 Oct 21;101(43):3320–2, 4–5.

Pettker CM, Grobman WA. Obstetric Safety and Quality. Obstetrics and gynecology. 2015 Jul;126(1):196–206.

Pyykonen A, Gissler M, Jakobsson M, Lehtonen L, Tapper AM. The rate of obstetric anal sphincter injuries in Finnish obstetric units as a patient safety indicator. European journal of obstetrics, gynecology, and reproductive biology. 2013 Jul;169(1):33–8.

Santos JV, Correia C, Cabral F, Bernardes J, Costa-Pereira A, Freitas A. Should European perinatal indicators be revisited? European journal of obstetrics, gynecology, and reproductive biology. 2013 Sep;170(1):85–9.

Sibanda T, Fox R, Draycott TJ, Mahmood T, Richmond D, Simms RA. Intrapartum care quality indicators: a systematic approach for achieving consensus. European journal of obstetrics, gynecology, and reproductive biology. 2013 Jan;166(1):23–9.

Simms RA, Ping H, Yelland A, Beringer AJ, Fox R, Draycott TJ. Development of maternity dashboards across a UK health region; current practice, continuing problems. European journal of obstetrics, gynecology, and reproductive biology. 2013 Sep;170(1):119–24.

Smit M, Chan KL, Middeldorp JM, van Roosmalen J. Postpartum haemorrhage in midwifery care in the Netherlands: validation of quality indicators for midwifery guidelines. BMC pregnancy and childbirth. 2014;14:397.

Smit M, Sindram SI, Woiski M, Middeldorp JM, van Roosmalen J. The development of quality indicators for the prevention and management of postpartum haemorrhage in primary midwifery care in the Netherlands. BMC pregnancy and childbirth. 2013;13:194.

Srinivas SK, Fager C, Lorch SA. Evaluating risk-adjusted cesarean delivery rate as a measure of obstetric quality. Obstetrics and gynecology. 2010 May;115(5):1007–13.

Thanh NX, Jacobs P, Wanke MI, Hense A, Sauve R. Outcomes of the introduction of the MOREOB continuing education program in Alberta. Journal of obstetrics and gynaecology Canada: JOGC = Journal d'obstetrique et gynecologie du Canada: JOGC. 2010 Aug;32(8):749–55.

Visser VS, de Groot CJ, Luitjes S, Wouters MG, van Lith J. Comparative analysis of recommendations in local Dutch guidelines on 'Hypertension and pregnancy'. Pregnancy hypertension. 2011 Apr;1(2):176–84.

Woiski MD, Hermens RP, Middeldorp JM, Kremer JA, Marcus MA, Wouters MG, et al. Haemorrhagia post partum; an implementation study on the evidence-based guideline of the Dutch Society of Obstetrics and Gynaecology (NVOG) and the MOET (Managing Obstetric Emergencies and Trauma-course) instructions; the Fluxim study. BMC pregnancy and childbirth. 2010;10:5.

Woiski MD, Scheepers HC, Liefers J, Lance M, Middeldorp JM, Lotgering FK, et al. Guideline-based development of quality indicators for prevention and management of postpartum hemorrhage. Acta obstetricia et gynecologica Scandinavica. 2015 Oct;94(10):1118–27.

Zeitlin J, Wildman K, Breart G, Alexander S, Barros H, Blondel B, et al. PERISTAT: indicators for monitoring and evaluating perinatal health in Europe. European journal of public health. 2003 Sep;13(3 Suppl):29–37.

## Supporting information

S1 File Sauvegrain et al_ Article POne. PRISMA 2009 checklist.
(DOC)
